# The Effectiveness of Full Pulpotomy Versus Root Canal Therapy for Mature Teeth With Irreversible Pulpitis: A Systematic Review and Meta-Analysis

**DOI:** 10.7759/cureus.114339

**Published:** 2026-08-11

**Authors:** Abdullah A Alhajeri, Salem J Salem, Yaser Atash, Fahad Alfadhli, Danah Alkhashan, Shekhah Alkhabbaz, Saba Almusabehi, Taiba Almutairi, Anfal Alkhulaifi, Hamzah Alkandari, Othman M Almobarak, Bashayer Alsahli, Eissa Alhemali, Masoud Almasoud

**Affiliations:** 1 Dentistry, Kuwait Institute for Medical Specialization, Al Ahmadi, KWT; 2 Department of Dentistry, Ministry of Health, Kuwait City, KWT; 3 Department of Dentistry, Taima Dental Center Polyclinic, Ministry of Health, Al Jahra, KWT; 4 Department of Dentistry, Rumaithiya Polyclinic, Kuwait City, KWT; 5 Department of Dentistry, Abdulrahman Alzaid Mishref West Clinic, Kuwait City, KWT

**Keywords:** full pulpotomy, mature permanent teeth, meta-analysis, root canal therapy, s: irreversible pulpitis, vital pulp therapy

## Abstract

Full pulpotomy preserves the radicular pulp and may reduce the procedural burden of root canal therapy (RCT), but comparative randomized evidence in mature teeth with irreversible pulpitis remains limited. We performed a systematic review and meta-analysis of randomized controlled trials comparing the two procedures. PubMed, Scopus, Web of Science, and Cochrane CENTRAL were searched from inception through February 2026. Five trials comprising 832 participants were eligible. The primary outcome was pain at seven days after treatment; secondary outcomes were pain at one day, time to pain relief, moderate-to-severe pain at seven days, clinical or radiographic success, and postoperative analgesic use. Risk of bias was assessed using Version 2 of the Cochrane Risk of Bias tool for randomized trials (RoB 2).

Pain at seven days did not differ significantly between full pulpotomy and RCT (mean difference (MD) = -0.31, 95% confidence interval (CI): -0.71 to 0.10; p = 0.14), and the likelihood of successful healing was similar ( risk ratio (RR) = 1.00, 95% CI: 0.81 to 1.24; p = 0.99). Pain at one day was lower after pulpotomy (MD = -1.60, 95% CI: -2.99 to -0.21; p = 0.02), although this association lost statistical significance when several individual studies were omitted. The pooled estimate for the variably defined reported time-to-relief outcome favored pulpotomy: RCT required 18.86 hours longer than pulpotomy (MD for RCT minus pulpotomy, 95% CI: 12.58 to 25.14; p < 0.001). Moderate-to-severe pain at seven days and analgesic use were not significantly different. On the basis of the available randomized evidence, full pulpotomy can achieve short-term pain and healing outcomes comparable with RCT in carefully selected mature permanent teeth. RCT remains the established reference treatment, whereas full pulpotomy may be considered a tissue-preserving alternative in appropriate cases. Further large-scale randomized trials using standardized outcome definitions and longer follow-up periods are warranted.

## Introduction and background

Root canal therapy (RCT) has long been the usual definitive treatment for mature permanent teeth with irreversible pulpitis because it removes the inflamed or infected tissue and enables subsequent disinfection and obturation of the root canal system [1]. [1]. When delivered under appropriate conditions, RCT is predictable; nevertheless, it is technically demanding, time-intensive, and often costly [2]. Access to specialist endodontic services, patient anxiety, financial constraints, and the feasibility of repeat appointments can also influence whether treatment is completed [3,4].

Full pulpotomy removes the coronal pulp while retaining potentially healthy radicular tissue. Interest in this approach has increased with the availability of calcium silicate biomaterials and a better understanding of the spatial variability of pulpal inflammation [5,6]. Evidence from biological and clinical studies suggests that portions of the pulp may remain viable and capable of repair after the source of irritation is removed and a durable coronal seal is achieved [7]. A recent randomized trial directly comparing full pulpotomy with RCT has further supported the evaluation of this less invasive strategy [8]. Comparative trials, however, differ in diagnostic criteria, operative protocols, pain scales, follow-up periods, and definitions of success. These differences have contributed to uncertainty about postoperative pain and treatment durability. We therefore conducted a systematic review and meta-analysis of randomized trials to compare the effectiveness of full pulpotomy with RCT in mature permanent teeth diagnosed with irreversible pulpitis.

## Review

Materials and methods

We conducted this systematic review and meta-analysis in accordance with the Preferred Reporting Items for Systematic Reviews and Meta-Analyses (PRISMA) [9] and the methodology outlined in the Cochrane Handbook of Systematic Reviews and Meta-Analyses [10].

Literature Search and Screening

PubMed, Scopus, Web of Science, and Cochrane CENTRAL were searched from database inception through February 2026. The search combined terms for pulpotomy or vital pulp therapy, root canal treatment, pulpitis, and randomized trials. Database-specific search strategies and yields are provided in Table 1. Reference lists of eligible reports were examined, and forward citation searching was performed to identify additional records. Titles and abstracts were screened before potentially eligible reports underwent full-text assessment.

**Table 1:** Detailed search strategy for each database

Database	Search keywords	Results
PubMed	(pulpotomy OR "vital pulp therapy" OR VPT OR "pulp capping") AND ("root canal therapy" OR "root canal treatment" OR RCT OR pulpectomy OR "endodontic treatment") AND (pulpitis OR "irreversible pulpitis" OR "symptomatic pulpitis" OR "pulp exposure") AND (randomized OR randomised OR "clinical trial" OR RCT)	N = 191
Scopus	TITLE-ABS-KEY ( pulpotomy OR "vital pulp therapy" OR vpt OR "pulp capping" ) AND TITLE-ABS-KEY ( "root canal therapy" OR "root canal treatment" OR rct OR pulpectomy OR "endodontic treatment" ) AND TITLE-ABS-KEY ( pulpitis OR "irreversible pulpitis" OR "symptomatic pulpitis" OR "pulp exposure" ) AND TITLE-ABS-KEY ( randomized OR randomised OR "clinical trial" OR rct )	N = 182
Web of Science	(pulpotomy OR "vital pulp therapy" OR VPT OR "pulp capping") AND ("root canal therapy" OR "root canal treatment" OR RCT OR pulpectomy OR "endodontic treatment") AND (pulpitis OR "irreversible pulpitis" OR "symptomatic pulpitis" OR "pulp exposure") AND (randomized OR randomised OR "clinical trial" OR RCT)	N = 107
Cochrane CENTRAL	(pulpotomy OR "vital pulp therapy" OR VPT OR "pulp capping") AND ("root canal therapy" OR "root canal treatment" OR RCT OR pulpectomy OR "endodontic treatment") AND (pulpitis OR "irreversible pulpitis" OR "symptomatic pulpitis" OR "pulp exposure")	N = 251

Eligibility Criteria

Studies were eligible if they (1) enrolled patients with irreversible pulpitis in mature permanent teeth; (2) compared full pulpotomy with RCT; and (3) used a randomized controlled trial design. Studies involving teeth without irreversible pulpitis, nonrandomized study designs, review articles, and conference abstracts were excluded.

Outcomes

The primary outcome was postoperative pain at seven days. Pain intensity was reported using a visual analog scale (VAS) or a numerical rating scale. The graphic rating method described by Hayes and Patterson is cited as the original source underlying the VAS concept [11]; a score of 0 denotes no pain, whereas the upper anchor denotes the worst pain. Secondary outcomes were pain at one day, moderate-to-severe pain at seven days, time to pain relief, clinical or radiographic treatment success, and postoperative analgesic use.

Risk-of-Bias Assessment

Risk of bias was evaluated using the Cochrane RoB 2 tool [12]. Judgments covered the randomization process, deviations from intended interventions, missing outcome data, outcome measurement, and selection of the reported result. Each domain and the overall study were classified as having low risk, some concerns, or high risk. Disagreements were resolved through discussion with the corresponding author.

Data Extraction

A standardized spreadsheet was used to record the study setting and design, recruitment and follow-up periods, group sizes, age and sex distributions, eligibility criteria, diagnostic definitions, outcomes, principal findings, RoB 2 judgments, and quantitative data needed for synthesis.

Statistical Analysis

For dichotomous endpoints, event counts and denominators were summarized as risk ratios (RRs) with 95% confidence intervals (CIs). Continuous outcomes were pooled as mean differences (MDs) with 95% CIs. Random-effects models were fitted using restricted maximum likelihood, consistent with the Stata output shown in the forest plots. Statistical heterogeneity was quantified using I²; values of 50% or greater were considered substantial, and the Cochran Q test was interpreted alongside I². When heterogeneity was substantial, leave-one-out analyses were undertaken by omitting one study at a time. Two-sided p-values below 0.05 were considered statistically significant. Analyses were performed in Stata MP version 19 (StataCorp, College Station, TX).

Results

Literature Search and Study Selection

**Figure 1:**
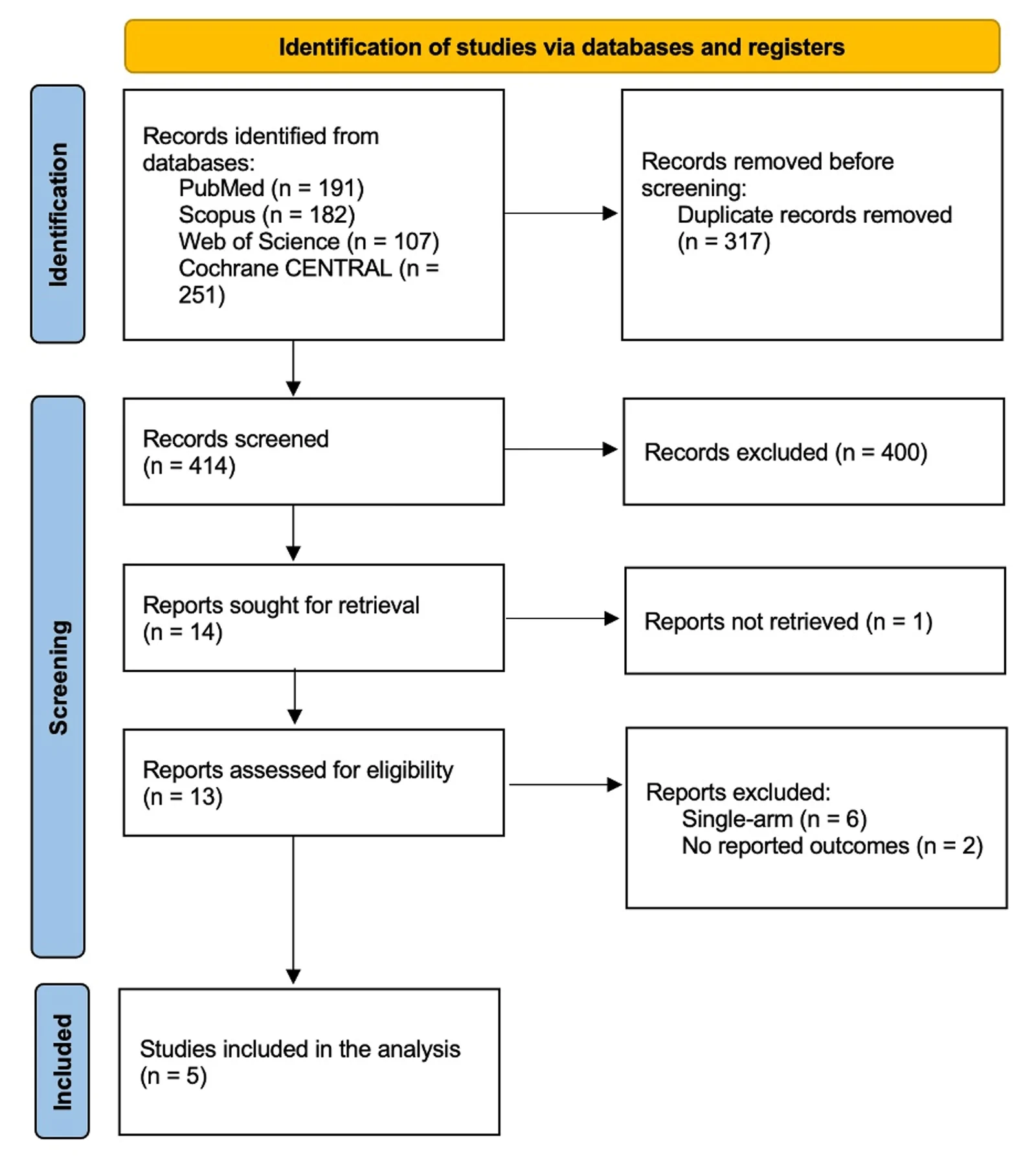
PRISMA flow chart depicting the study selection PRISMA: Preferred Reporting Items for Systematic Reviews and Meta-Analyses

The search identified 731 records. After removal of 317 duplicates, 414 records were screened, and 400 were excluded at the title and abstract stage. Fourteen reports were sought for retrieval; one was unavailable, and 13 full texts were assessed. Eight reports were excluded (six single-arm studies and two reports without eligible outcomes), leaving five randomized trials for quantitative synthesis (Figure 1) [8,13-16].

Study Characteristics and Risk of Bias

The five trials were conducted in Iran, the United Kingdom, Turkey, Jordan, and China and enrolled patients between 2008 and 2023. In total, 832 participants were randomized: 424 (50.9%) to RCT and 408 (49.1%) to full pulpotomy. The sample included 326 males (39.2%), and the reported mean ages ranged from 24.63 to 38.32 years across the groups. Table 2 presents the study-level characteristics, eligibility criteria, outcomes, and main findings.

**Table 2:** Summary and baseline characteristics of the included studies RCT: root canal therapy; SD: standard deviation; EPT: electric pulp test; DEJ: dentin-enamel junction; VAS: visual analog scale; FP: full pulpotomy; NSAIDs: nonsteroidal anti-inflammatory drugs; AAE: American Association of Endodontists; NRS: numerical rating scale

Variable	Asgary and Eghbal, 2010 [16]		Patel et al., 2025 [15]		Sarı et al., 2024 [14]		Taha et al., 2023 [13]		Zhu et al., 2024 [8]	
	RCT	Pulpotomy	RCT	Pulpotomy	RCT	Pulpotomy	RCT	Pulpotomy	RCT	Pulpotomy
Country	Iran		UK		Turkey		Jordan		China	
Design	Multicenter, noninferiority, randomized, parallel-group, open-label RCT		Multicentre, single-blinded RCT		Double-arm, prospective RCT (parallel groups)		Parallel, double-blind RCT		Single-blinded, single-center RCT	
Timeframe	Apr–Sep 2008; 12-week follow-up		Sep 2019–Jul 2022; 7-day primary (24-month secondary)		Jan–Oct 2023; 1-week follow-up (1-year planned)		Apr 2019–Jan 2020; 12-month follow-up		2022; 12-month follow-up	
Sample size	202	205	80	61	32	32	30	30	80	80
Age, years, mean (SD)	26 ± 8	27 ± 8	Mean 37 (median 34.8); range 18–75 (per group NR)	Mean 37 (median 34.8); range 18–75 (per group NR)	28.6 ± 8.55	24.63 ± 7.38	33.53 ± 9.69	25.97 ± 10.23	38.32 ± 11.89	34.20 ± 9.85
Male, n (%)	82 (40.6%)	72 (35.1%)	41 (51.3%)	27 (44.3%)	16 (50.0%)	15 (46.9%)	12 (40.0%)	18 (60.0%)	23 (28.8%)	20 (25.0%)
Female, n (%)	120 (59.4%)	133 (64.9%)	39 (48.8%)	34 (55.7%)	16 (50.0%)	17 (53.1%)	18 (60.0%)	12 (40.0%)	57 (71.3%)	60 (75.0%)
Inclusion criteria	Vital molar (radicular pulp bleeding confirmed after coronal pulp amputation)		Age ≥16 years, good general health		Age 18–50 years, no systemic disease		Irreversible pulpitis symptoms (spontaneous pain, hot/cold exacerbation, radiating pain)		Molar with deep caries exposing vital pulp (cold spray/EPT + visual hemorrhage confirmed)	
	Irreversible pulpitis symptoms (spontaneous pain, hot/cold exacerbation, radiating pain)		Symptomatic irreversible pulpitis requiring endodontic treatment		Mandibular 1st/2nd molars with complete root development		No pulp necrosis signs (no sinus tract or swelling)		Spontaneous/radiating pain; hot/cold pain lasting minutes after stimulus removal	
	Opted for extraction for pain relief		Positive response to thermal stimulation		Probing pocket depth ≤ 3 mm, mobility within normal limits		Tooth in occlusion; restorable via direct restoration		No prominent periapical or furcation radiolucency	
	Age 9–65 years		Molar teeth		Deep/extremely deep caries on periapical radiograph		Probing pocket depth and mobility within normal limits		Age 18–50 years	
	Willing to attend follow-up; written informed consent				Moderate or severe pulpitis (Wolters classification)		Radiographically: deep caries ≥ 2/3 DEJ-to-pulp or pulp exposure; mature roots, closed apices		Able to attend follow-up; written informed consent	
					Vital pulp, positive cold test		Age 15–50 years			
					Preoperative VAS pain > 4 mm					
Exclusion criteria	Moderate or severe marginal periodontitis		Fistula or swelling present		Antibiotic therapy in the last 3 months		Bleeding uncontrolled after full pulpotomy within 10 min		Uncontrolled bleeding after FP > 10 min, or invisible bleeding from ≥ 1 orifice	
	Non-restorable tooth		Anterior teeth with aesthetic concerns		NSAIDs use within the last 12 hours		Invisible bleeding from ≥1 orifice (indicating necrosis)		Non-restorable tooth	
	Internal or external root resorption		External or internal root resorption		Diabetes, immunosuppressive disease, or pregnancy				Periodontal disease (probing pocket depth > 3 mm)	
	Root canal calcification		Multiple carious teeth in the same quadrant		Non-restorable tooth or requiring post-core				Crack fracture; internal/external root resorption; pulp calcification	
	Active systemic disease; physical/mental disability; pregnant/nursing		Mobile teeth; pregnant women; age < 16; unable to consent		Devital pulp/no cold test response				Pregnant/nursing; physically/mentally disabled	
			Antibiotics in the previous month; immunocompromised		No pulp exposure after caries removal					
			Incisor or premolar teeth		Hemostasis not achievable within 8 min (converted to RCT)					
Clinical diagnosis	Irreversible pulpitis, permanent molars (vital pulp confirmed; spontaneous pain, hot/cold exacerbation)		Symptomatic irreversible pulpitis, molars (prolonged hot/cold pain > 20 s or spontaneous; tenderness to percussion)		Irreversible pulpitis, mandibular mature molars (AAE + Wolters: moderate or severe); preoperative VAS > 4 mm		Symptomatic irreversible pulpitis, mature molars (carious exposure; mean NRS 7.5–7.9/10)		Irreversible pulpitis, mature molars (carious exposure; spontaneous/radiating pain; mean NRS: FP 8.0, RCT 7.8)	
Assessed outcomes	Primary: Clinical/radiographic success at 6 mo, 1, 2, 5 years. Secondary: Postoperative pain (NRS 0–9) at baseline, 6–60 h, days 3–7; sum of pain intensity (SPI); percussion pain at days 1 and 7; analgesic consumption		Primary: Postoperative pain (VAS 0–100 mm) at baseline, days 1, 3, 5, 7. Secondary: HRQoL (EQ-5D-5L); OHRQoL (OHIP-14); analgesic use; clinical/radiographic assessment (PA + CBCT) at baseline, 7 days, 12 and 24 mo		Postoperative pain (VAS 0–10) at preoperative, 6, 12, 24, 48, 72 h, 7 days; hemostasis time; Wolters classification subgroup comparison; clinical exam at 1 wk		Postoperative pain (NRS 0–10) at preoperative, days 1–3, 5, 7; analgesic use; clinical/radiographic success at 6 and 12 mo; QoL (OHIP-17); patient satisfaction		Postoperative pain (NRS 0–10) preoperatively and daily days 1–7; clinical/radiographic success at 3, 6, 12 mo; pulp sensibility (EPT) at 3, 6, 12 mo (FP only); treatment time and cost	
Key findings	Pulpotomy (PCEM) noninferior to ORCT. Median time to pain-free: PCEM 18 h vs. ORCT 36 h (P < 0.01). Mean PI over 7 days: ORCT 1.26 vs. PCEM 0.67 (p < 0.001). Percussion pain lower in PCEM at days 1 and 7 (p < 0.001). Analgesic use: ORCT 59% vs. PCEM 36% (p < 0.001).		Pulpotomy equivalent to RCT in pain reduction (VAS AUC 221.55 vs. 243.9, p = 0.804). Pain decreased significantly in both groups over 7 days (p < 0.001). PRCT (converted pulpotomy) had higher pain than pulpotomy (p = 0.045) and RCT (p = 0.049). Pulpotomy had shorter procedure time (73.8 vs. 100.3 min) and improved HRQoL in all groups.		Moderate pulpitis: no significant TP vs. RCT difference at any time point (p > 0.05); TP had faster relief (significant decrease at 12 h vs. 24 h for RCT). Severe pulpitis: TP had significantly lower pain at 24 h and 72 h (p < 0.05). Preoperative pain correlated with hemostasis time (r = 0.564, p = 0.012). 3 TP patients required RCT at 13, 17, and 20 days post-treatment.		1-year success: pulpotomy 93% vs. RCT 93%. Day-1 pain lower after pulpotomy (3.03 vs. 4.90, p = 0.037). Fewer analgesics: pulpotomy 20% vs. RCT 46.7% (p = 0.028). QoL improved in both groups (p = 0.618). Patient satisfaction was higher for pulpotomy in time, intraoperative pain, pleasantness, and cost (p < 0.05).		12-month success: FP clinical 97.3% vs. RCT 98.6%; radiographic 93.3% vs. 94.6% (p > 0.05). Day-1 pain: FP NRS 2.3 vs. RCT 5.5 (p = 0.031). Treatment time: FP 43.5 min vs. RCT 112.5 min (p = 0.020). Cost: FP RMB 882 vs. RCT RMB 1985.5 (p = 0.022).	

**Figure 2:**
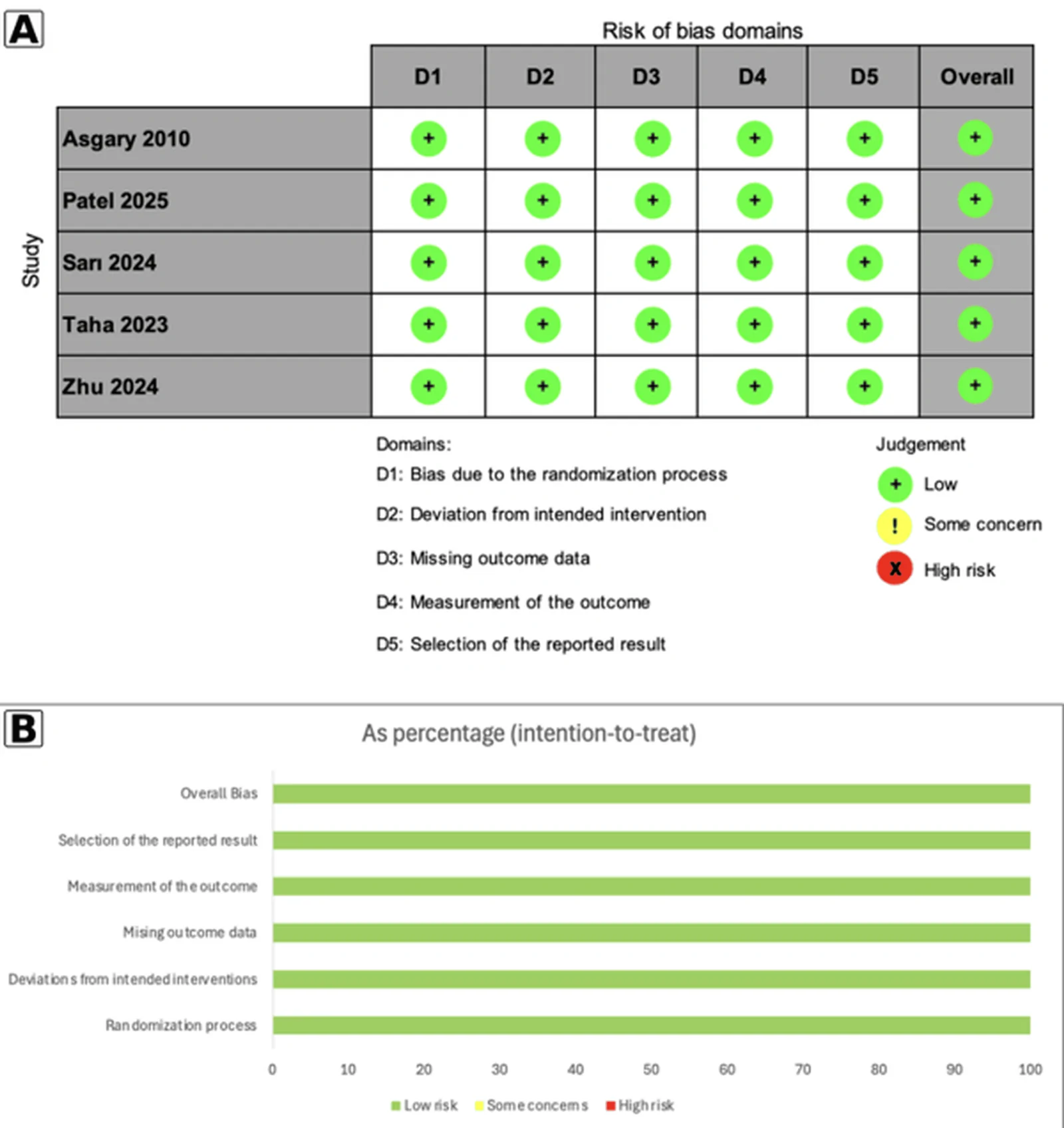
Risk of bias assessment for the included randomized controlled trials using RoB 2 tool (A) Traffic-light plot showing domain-level judgments for each trial. (B) Summary plot showing the distribution of judgments across all trials Asgary and Eghbal [16], Patel et al. [15], Sarı et al. [14], Taha et al. [13], and Zhu et al. [8] RoB 2: Version 2 of the Cochrane Risk of Bias tool for randomized trials

All five trials were assessed as having an overall low risk of bias according to the RoB 2 tool (Figure 2).

Primary Outcome

**Figure 3:**
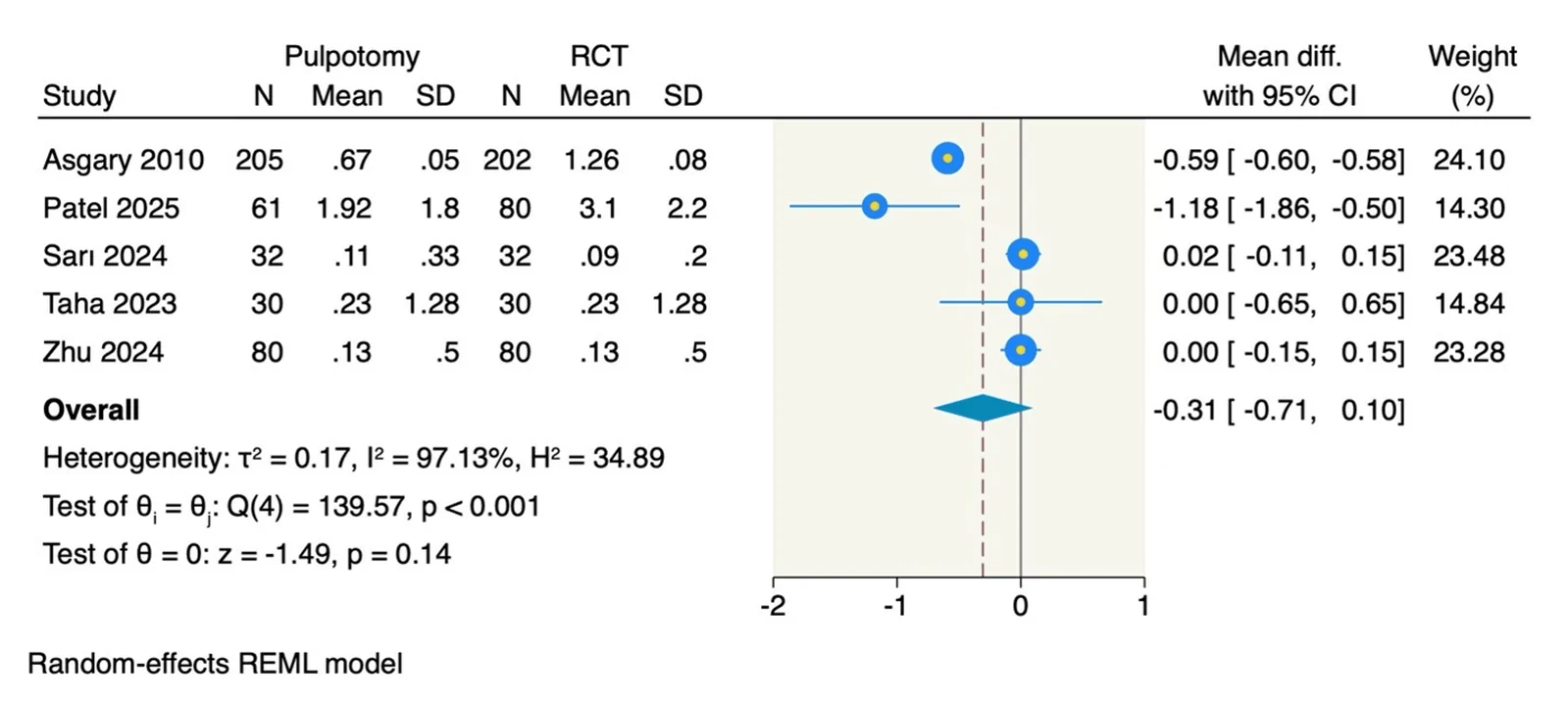
Random-effects model of pain score assessment at seven days postoperatively Asgary and Eghbal [16]; Patel et al. [15]; Sarı et al. [14]; Taha et al. [13]; Zhu et al. [8] RCT: root canal therapy; SD: standard deviation; CI: confidence interval; REML: restricted maximum likelihood

**Figure 4:**
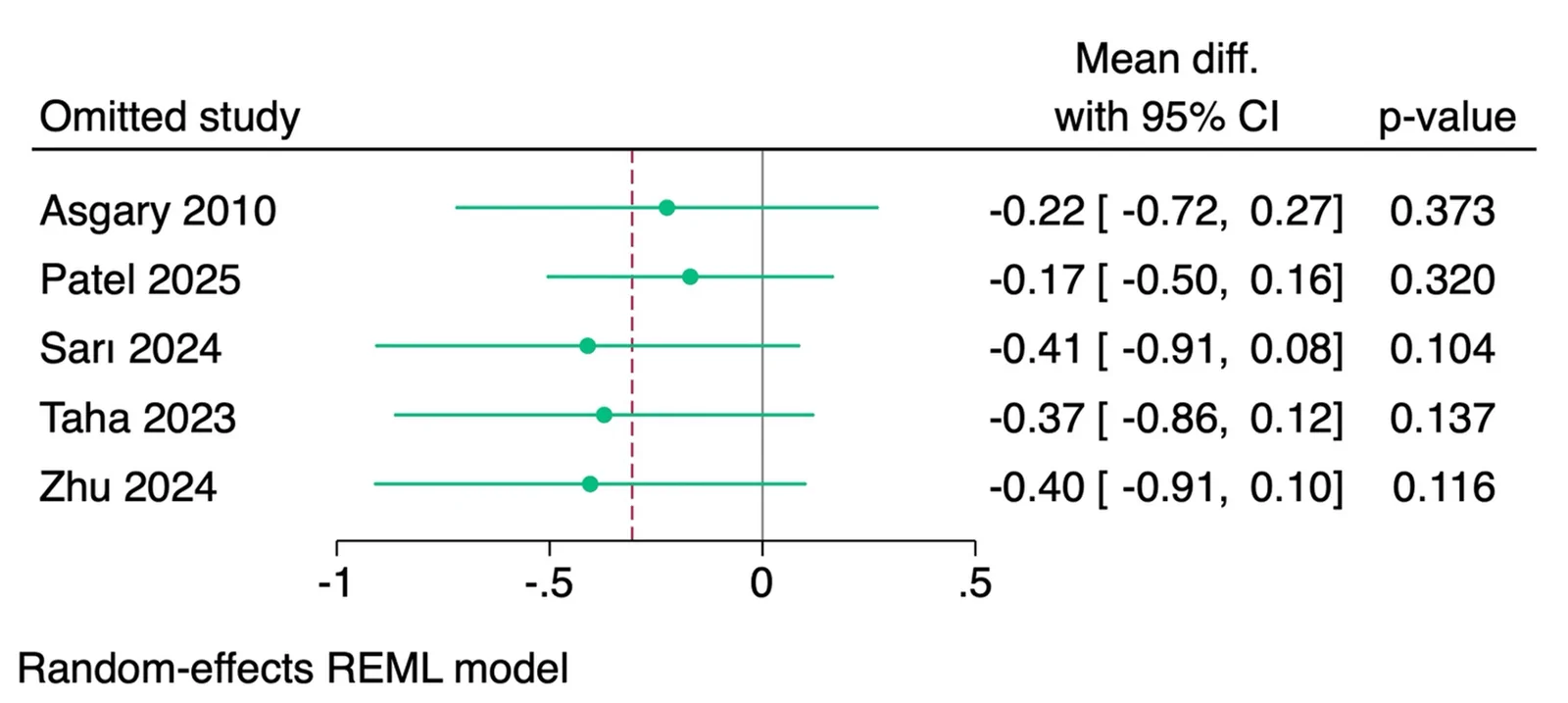
Leave-one-out sensitivity analysis of pain score assessment at seven days postoperatively Asgary and Eghbal [16]; Patel et al. [15]; Sarı et al. [14]; Taha et al. [13]; Zhu et al. [8] CI: confidence interval; REML: restricted maximum likelihood

All five trials reported pain at seven days [8,13-16]. The pooled MD was -0.31 (95% CI: -0.71 to 0.10; p = 0.14), indicating no statistically significant difference between full pulpotomy and RCT. Heterogeneity was substantial (I² = 97.13%; Q-test p < 0.001) (Figure 3). Omitting one trial at a time did not materially alter the absence of a statistically significant difference (Figure 4).

Secondary Outcomes

**Figure 5:**
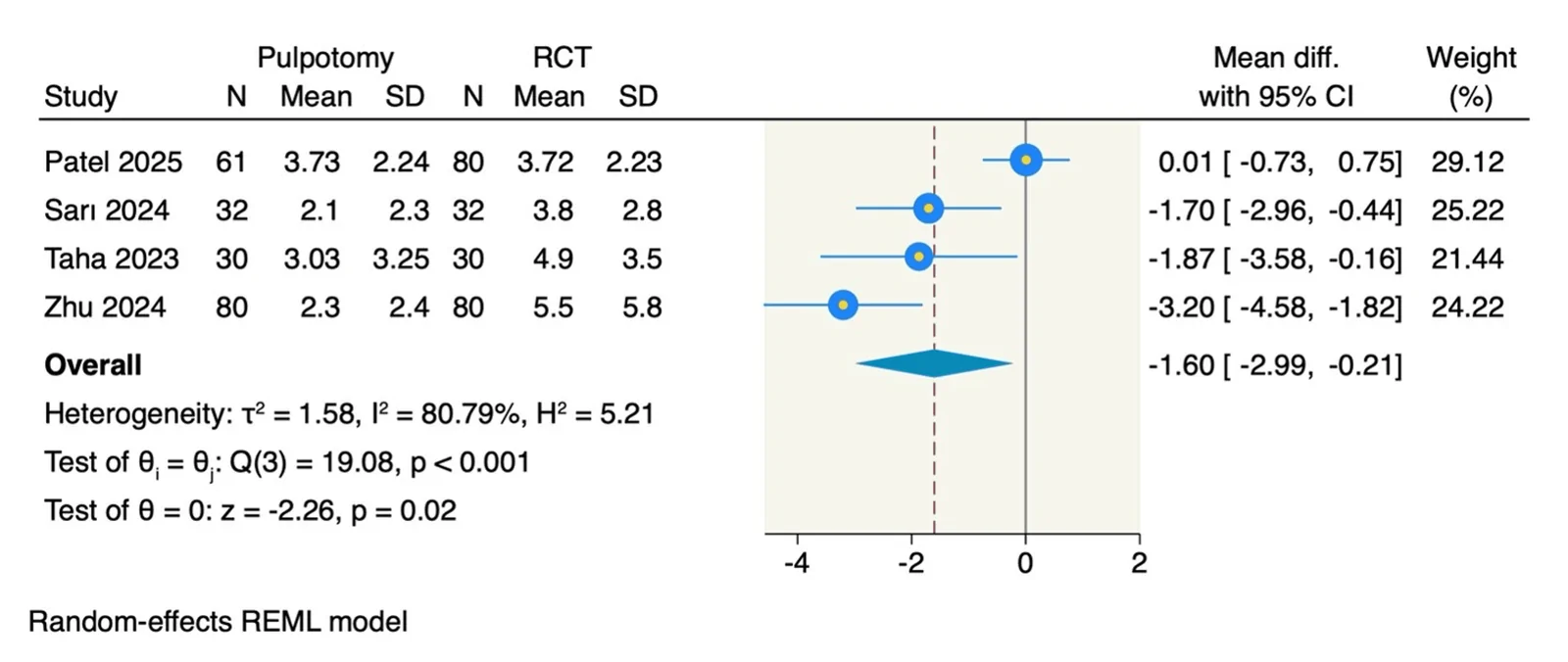
Random-effects model of the pain score assessment at one day postoperatively Patel et al. [15]; Sarı et al. [14]; Taha et al. [13]; Zhu et al. [8] RCT: root canal therapy; SD: standard deviation; CI: confidence interval; REML: restricted maximum likelihood

**Figure 6:**
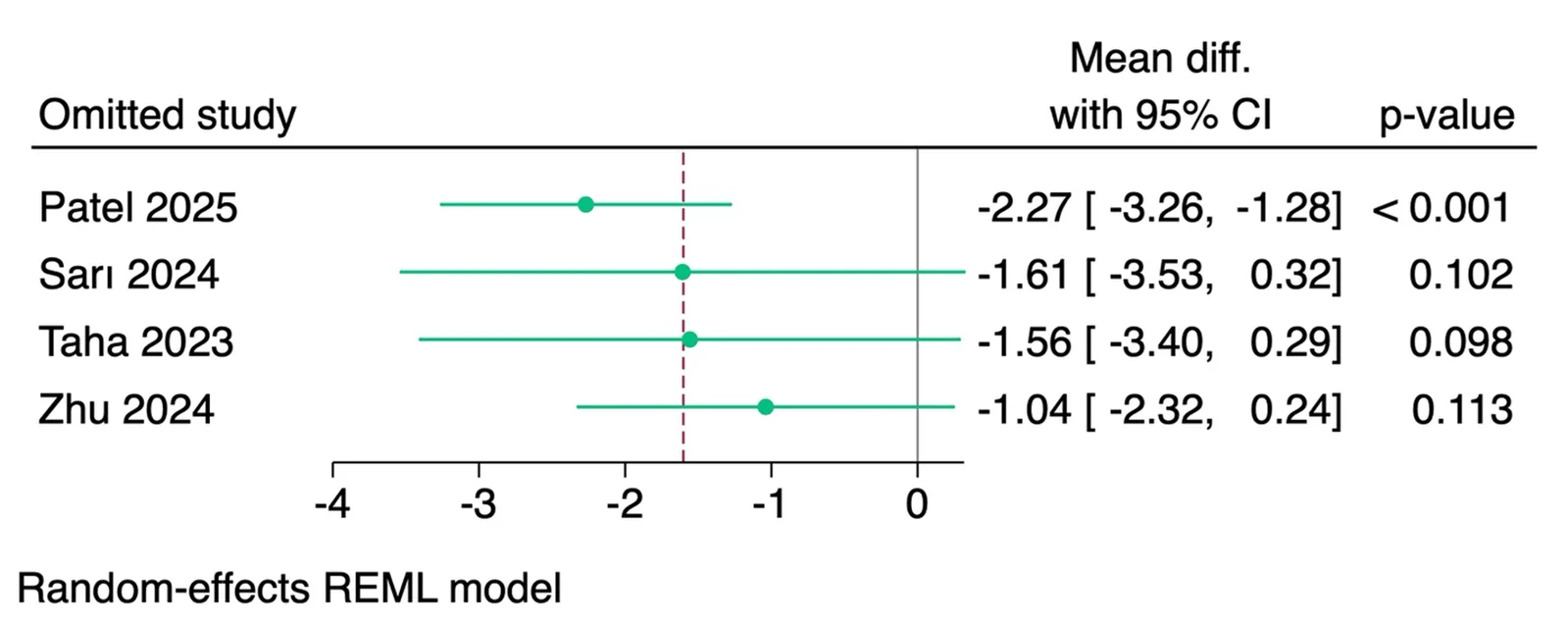
Leave-one-out sensitivity analysis of pain scores assessment at one day postoperatively Patel et al. [15]; Sarı et al. [14]; Taha et al. [13]; Zhu et al. [8] CI: confidence interval; REML: restricted maximum likelihood

Four trials reported pain at one day [8,13-15]. Full pulpotomy was associated with lower pain (MD = -1.60, 95% CI: -2.99 to -0.21; p = 0.02), with substantial heterogeneity (I² = 80.79%; p < 0.001) (Figure 5). In the leave-one-out analysis, the result was no longer statistically significant after exclusion of Sarı et al. [14], Taha et al. [13], or Zhu et al. [8] (Figure 6).

**Figure 7:**
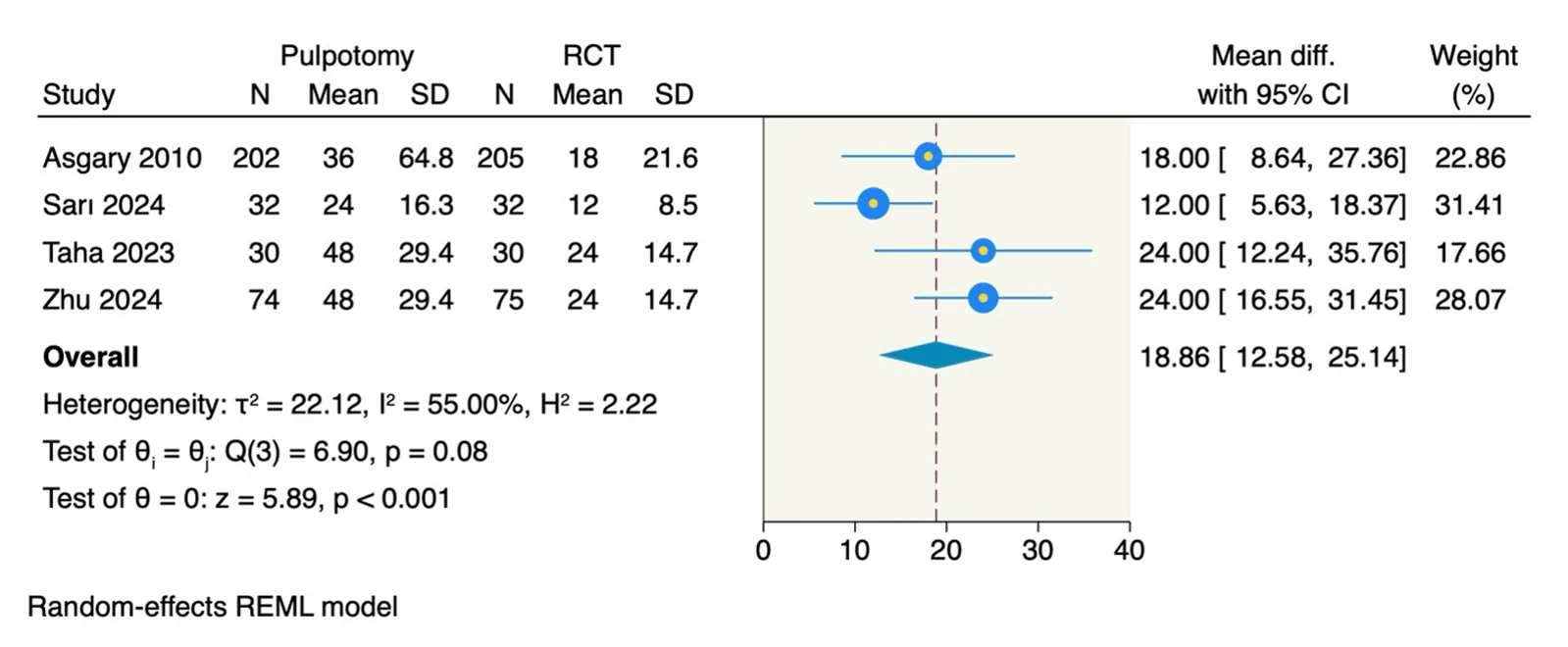
Random-effects model of the time needed to heal Asgary and Eghbal [16]; Sarı et al. [14]; Taha et al. [13]; Zhu et al. [8] RCT: root canal therapy; SD: standard deviation; CI: confidence interval; REML: restricted maximum likelihood

Four trials contributed to the time-to-relief analysis [8,13,14,16]. With the effect defined as RCT minus pulpotomy, the pooled estimate indicated that relief took 18.86 hours longer after RCT (95% CI: 12.58 to 25.14; p < 0.001; I² = 55%; Q-test p = 0.08) (Figure 7).

**Figure 8:**
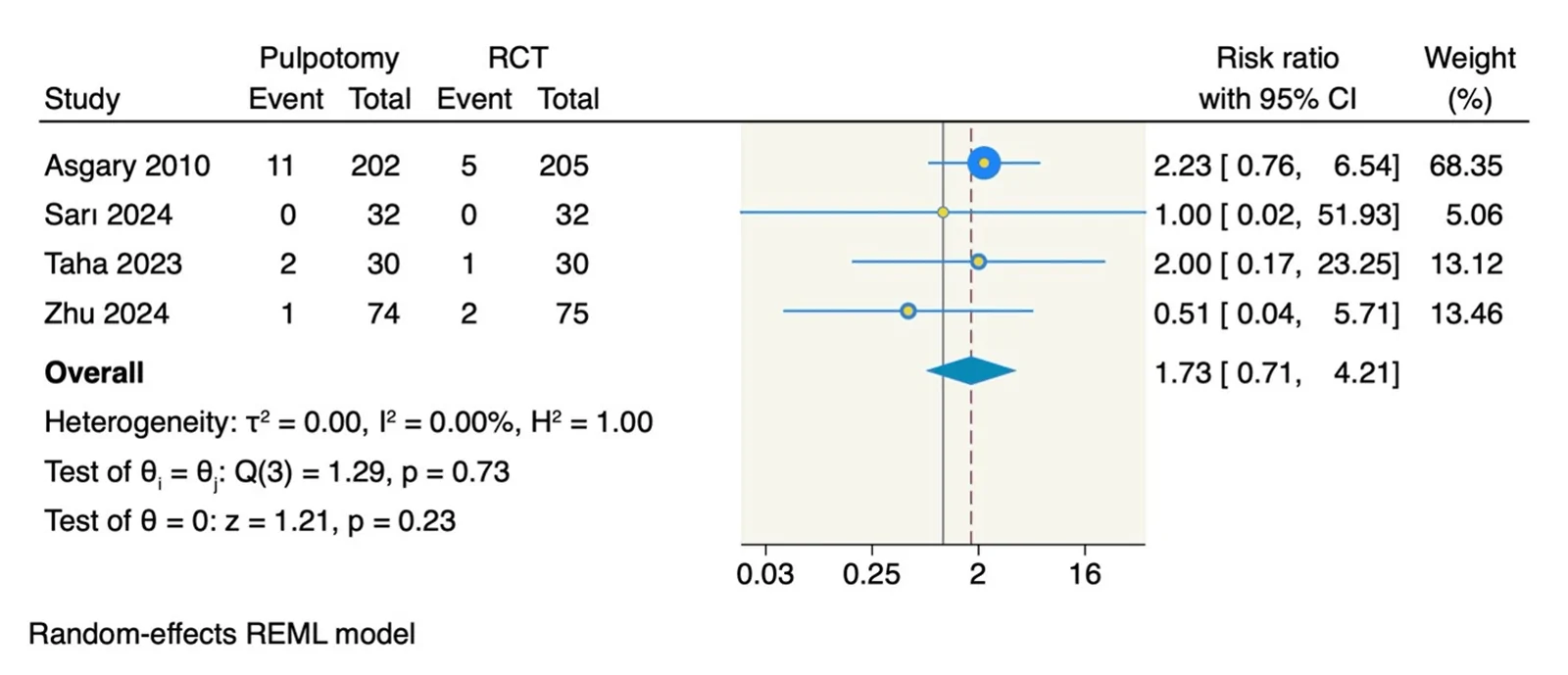
Random-effects model of the rate of moderate to severe pain following seven days Asgary and Eghbal [16]; Sarı et al. [14]; Taha et al. [13]; Zhu et al. [8] RCT: root canal therapy; CI: confidence interval; REML: restricted maximum likelihood

**Figure 9:**
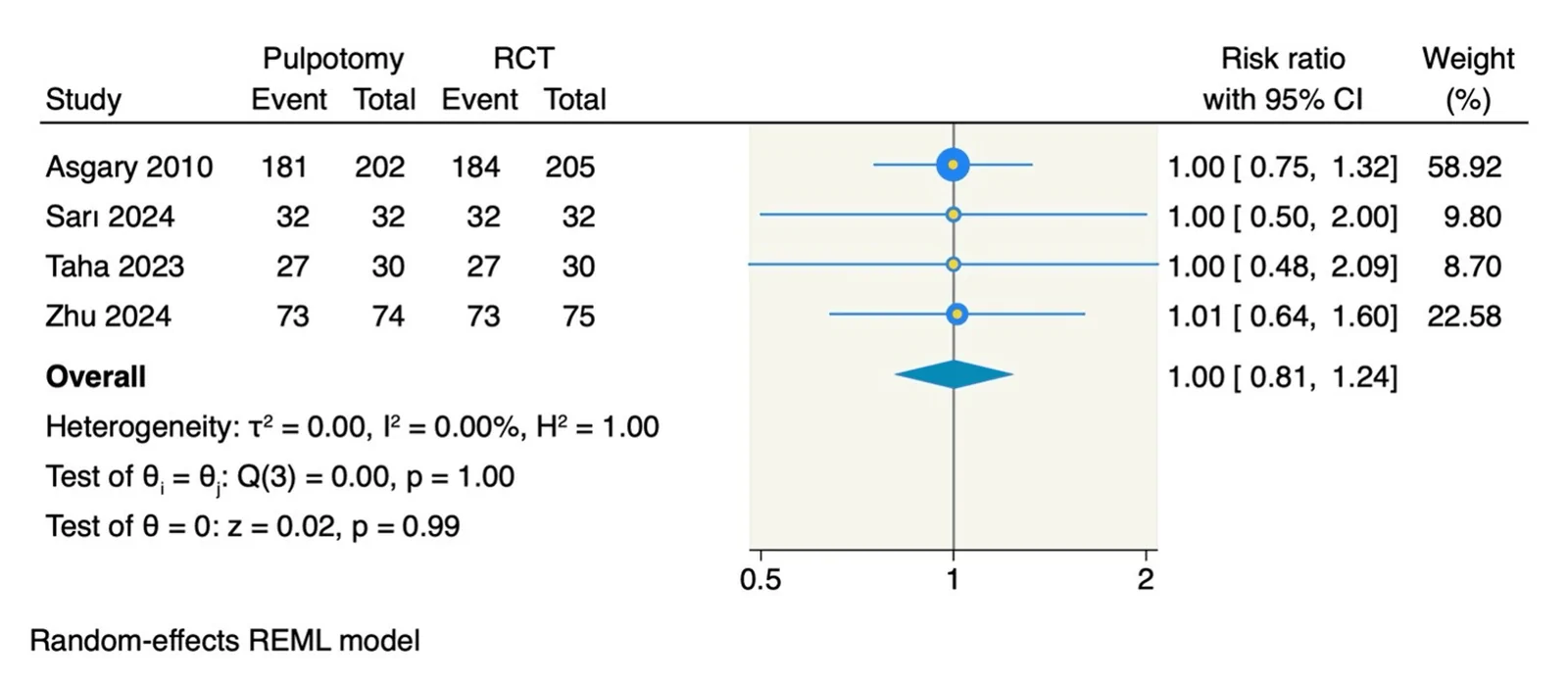
Random-effects model of the number of patients with healed pulpitis Asgary and Eghbal [16]; Sarı et al. [14]; Taha et al. [13]; Zhu et al. [8] RCT: root canal therapy; CI: confidence interval; REML: restricted maximum likelihood

**Figure 10:**
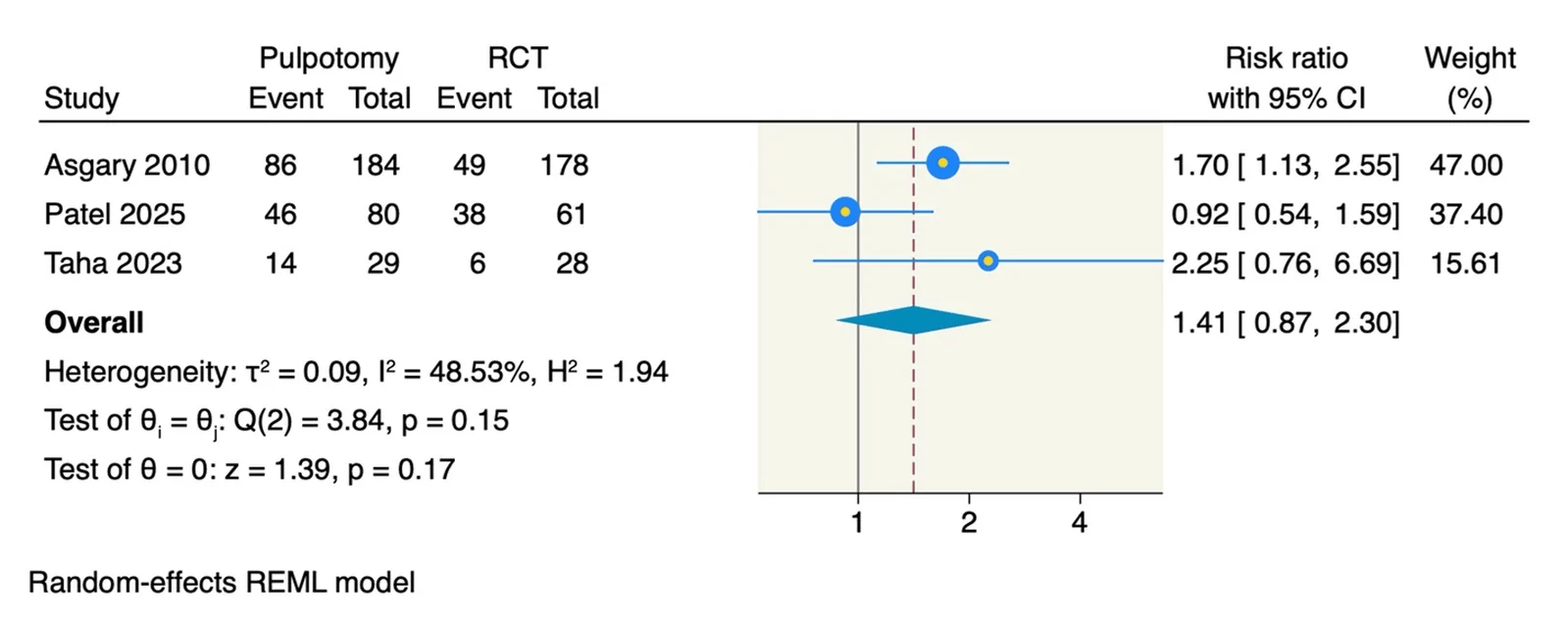
Random-effects model of the number of patients who required additional analgesics Asgary and Eghbal [16]; Sarı et al. [14]; Taha et al. [13]; Zhu et al. [8] RCT: root canal therapy; CI: confidence interval; REML: restricted maximum likelihood

Moderate-to-severe pain at seven days was reported in four studies [8,13,14,16]. Events occurred in 14 of 338 patients (4.1%) after RCT and eight of 342 patients (2.3%) after pulpotomy. With the RR defined as RCT divided by pulpotomy, the pooled comparison was not significant (RR = 1.73, 95% CI: 0.71 to 4.21; p = 0.23) (Figure 8). Four trials reported successful healing [8,13,14,16], with rates of approximately 92.5% in both groups and no between-group difference (RR for RCT versus pulpotomy = 1.00, 95% CI: 0.81 to 1.24; p = 0.99) (Figure 9). Three trials reported postoperative analgesic use [13,15,16]. Analgesics were used by 146 of 293 patients (49.8%) after RCT and 93 of 267 patients (34.8%) after pulpotomy; the pooled RR for RCT versus pulpotomy was 1.41 (95% CI: 0.87 to 2.30; p = 0.17), indicating no statistically significant difference (Figure 10).

Discussion

This synthesis of five randomized trials found that full pulpotomy and RCT produced comparable pain at seven days and similar clinical or radiographic success. Neither moderate-to-severe pain at seven days nor postoperative analgesic use differed significantly. The pooled time-to-relief estimate favored pulpotomy, whereas the day-one pain estimate also favored pulpotomy but was not stable in sensitivity analysis. These findings support full pulpotomy as a possible alternative for carefully selected mature teeth rather than as a universal substitute for RCT.

The conventional response to a diagnosis of irreversible pulpitis has been complete pulp extirpation because inflammation was assumed to be diffuse and irreversible [17,18]. The randomized evidence summarized here does not show a disadvantage to retaining radicular pulp with respect to short-term pain or the observed healing rate. Pain is a central patient-reported outcome in endodontics [19], and the lack of a meaningful difference at seven days suggests that recovery is acceptable with either approach. The success rates reported by the included trials were above 90% in both groups [8,13,14]. Pulpotomy may also shorten treatment, simplify delivery, and reduce costs in suitable cases [20,21], features that could be relevant when specialist access or multiple visits are difficult.

The apparent day-one advantage of pulpotomy may be related to less canal instrumentation and less mechanical irritation of the periapical tissues [15,22]. Nevertheless, substantial heterogeneity and loss of significance in several leave-one-out analyses limit confidence in this early effect. Variability in baseline pain, anesthetic and analgesic protocols, pain scales, and operative techniques may explain part of the inconsistency.

The biological rationale for vital pulp therapy has also evolved. Pulpitis is not always histologically uniform throughout the coronal and radicular pulp [23,24]. After caries and contaminated tissue are removed, residual radicular tissue may retain vascular, neural, immune, and reparative functions. Calcium silicate-based materials such as mineral trioxide aggregate and newer bioceramics provide sealing, bioactivity, and a favorable environment for hard-tissue repair [25,26]. By contrast, RCT necessarily removes all pulp tissue and enlarges the canal space; the loss of tooth structure associated with endodontic and restorative procedures can affect fracture resistance [27]. Preserving viable tissue where clinically appropriate is therefore consistent with minimally invasive endodontics and may maintain pulpal defense and proprioceptive functions [28].

The high heterogeneity in pain outcomes remains important. The trials used different definitions of irreversible pulpitis, biomaterials, hemostasis thresholds, restorative protocols, operators, and follow-up schedules. The pooled time-to-relief outcome should also be interpreted cautiously because the contributing studies did not use fully standardized timing definitions. These clinical differences restrict the certainty with which a single pooled estimate can be applied to every patient.

Recent professional statements increasingly acknowledge a role for vital pulp therapy in mature permanent teeth [29,30]. Expert consensus suggests that full pulpotomy may be considered when the tooth is restorable, the pulp remains vital, bleeding can be controlled, and an appropriate bioactive material and coronal seal can be provided [31]. RCT nevertheless remains the reference treatment in many settings because its indications, technique, and long-term evidence base are more established [18]. Choice of treatment should therefore reflect symptoms, pulpal and periapical status, restorability, operator experience, patient preferences, and the ability to provide follow-up.

Limitations

This review has several limitations that should be considered when interpreting the findings. Only five trials were included, which limits precision and prevents a reliable assessment of small-study effects. Pain-related analyses showed substantial statistical and clinical heterogeneity. Follow-up duration, diagnostic criteria, biomaterials, success definitions, and procedural protocols were not uniform. The time-to-relief endpoint was not defined identically across studies, and long-term outcomes were incompletely represented in the pooled analyses. Clinical diagnosis may not correspond perfectly to histologic inflammation, creating potential misclassification. Operator expertise was not consistently described, and the geographic concentration of the trials may reduce generalizability. Future multicenter randomized trials should use standardized diagnostic definitions, prespecified core outcomes, consistent pain scales, transparent reporting of group-level data, and longer follow-up.

## Conclusions

Available randomized evidence indicates that full pulpotomy can provide pain and healing outcomes broadly comparable to those of RCT in selected mature permanent teeth with irreversible pulpitis. RCT remains a dependable and established treatment, while full pulpotomy offers a less invasive option that preserves radicular pulp. More rigorous long-term trials are required before the indications and durability of pulpotomy can be defined with greater certainty.
